# Meshing Mechanism and Simulation Analysis of Silent Chain Based on Rigid-Flexible Intelligent Dynamics Technology

**DOI:** 10.1155/2022/3346641

**Published:** 2022-04-13

**Authors:** Song Ding

**Affiliations:** Changchun Normal University College of Engineering, Changchun Jilin 130032, China

## Abstract

Silent chain is widely used in the field of mechanical transmission. Compared with ordinary chain transmission, it has prominent advantages such as high precision, low noise, and being suitable for high speed and heavy loads. Based on the design concept of intelligent dynamics, the phase variation is carried out. The multiphase transmission silent chain retains the original advantages of the single-phase transmission silent chain, and its structure bearing capacity is improved, the polygon effect is reduced, and the fluctuation, noise, and vibration of elastic edge are improved. Based on rigid-flexible intelligent dynamics technology, the transmission characteristics of a multiphase transmission silent chain system were studied. In this paper, the meshing contact force between the chain plate and the sprocket is described in detail, and the 3d model and dynamic model of the multiphase transmission silent chain are established. Topological optimization method was used to design the lightweight of the multiphase transmission silent chain, and the existing target of lightweight was realized. The dynamics simulation was carried out to extract the dynamics data and analyze the dynamics characteristics such as tight edge fluctuation, instantaneous transmission ratio, and meshing contact force. The results show that the model correctly predicts the dynamic behavior of the system, and the high speed inhibits the lateral fluctuation of the system. The peak value of meshing contact force increases with the increase of rotational speed, and the multiphase drive can be used in practical control system design.

## 1. Introduction

With the development of spacecraft, robots, and other mechanical systems towards the direction of light, high speed, and high precision, a large number of rigid-flexible coupling systems with light, flexible components appear. Previous control studies on such rigid-flexible coupled systems are mostly based on the traditional zero-order approximate dynamics model [1]. The silent chain consists of a series of silent chain plates and guide plates interlaced with pins and other forms of articulated assembly. Because of the structural characteristics of silent chain, compared with gear transmission, the silent chain is more convenient in the transmission process of large center distance and heavy load. Compared with the toothed belt drive, the silent chain will not slip, and the drive is more reliable. Compared with roller chain drive, hinge wear is not serious and can maintain accurate transmission accuracy. Compared with sleeve chain, the silent chain has higher movement accuracy and no noise during sleeve movement [2]. Silent chain noise is small. When a plurality of working chain plate silent chain is damaged, the silent chain will not break, and the work of the host will not be affected and repair damaged chain plate normal work. If the working load needs to increase, the tooth shape chain can increase the chain plate and add a small size in the width direction. The pin shaft and sleeve of the sleeve chain constitute a reciprocating swing hinge pair at work. When wear occurs, the chain elongates, leading to errors, jumping teeth, impact load, and even early failure. Through the flexible treatment of chain plate and pin shaft, scholars analyzed the stress in the meshing process of chain plate. The wear of the working chain plate of the silent chain is relatively uniform, so it can maintain good transmission accuracy [3]. In the mechanical field of many transmission modes, the application of silent chains is becoming more and more extensive, and superior transmission performance is becoming more and more prominent.

With the improvement of computer computing ability, finite element model, multibody dynamics model, and rigid-flexible coupling dynamics model are gradually used by scholars. The finite element model has also made many achievements in the study of vibration characteristics of the fixed-axis gear system, such as gear cracks, pitting corrosion, and broken teeth, but its calculation results are greatly affected by mesh accuracy, and the simulation time is long [4]. The simulation time of the multibody dynamic model is much better than that of the finite element model, but the calculation results are not ideal because the flexibility of some components is neglected. The coupled model between finite element model and dynamics model, considering both the flexibility of some important parts and simplifying some minor factors, makes the simulation time and get a better computational precision balance; the current study of gear system fault vibration characteristics is more focused on not considering speed fluctuation condition, although it has gained great research achievements. However, there are still shortcomings. The long-waist-type datum bore chain is a new type of variable tooth chain product, which can meet the “personalized” needs of different hosts by optimizing the combination of internal and external meshing + external meshing arrangement rules. The silent chain is a new type of transmission element with high efficiency, low noise, and strong wear resistance in the field of transmission, which can realize power and motion transmission under bad working conditions [5]. Chain transmission technology to design various host “personalized” demands of high-end tooth shape chain products, the chain product model, the structure of tooth shape chain products, and meshing mechanism in the design of continuous variation, innovation, and upgrade superior performance and stable transmission. The design, manufacture, and production of toothed chain have world-class technical level [6]. Enterprises producing silent chain products mainly rely on surveying and mapping and imitation of foreign products, resulting in large vibration and noise; transmission performance is not up to standard. In the process of power transmission and motion, the polygon effect is weakened, and transverse fluctuation decreases. Although it has significant advantages and can solve the impact and vibration problems existing in the current silent chain, it lacks the support of necessary theoretical derivation, simulation analysis, and experimental data. In order to meet the needs of engineering applications, how to accurately predict the impact of the coupling between the rigid body motion and the chain on the dynamic response of the whole system is one of the urgent problems in the field of mechanics.

## 2. Related Work

Chains have been used for centuries for mechanical transmission and for conveying items on conveyors and elevators. Throughout the twentieth century, the carrying capacity and speed of silent chains increased significantly due to advances in material quality, processing technology, and chain design technology. Chain drive technology is constantly updated, and domestic and foreign scholars to its in-depth exploration and research. Deng et al. proposed a comprehensive program to establish the chain drive multibody model based on the minimum information given by users, which realized the initialization of the position and speed of chain drive components and confirmed the appropriate initial conditions. The initial conditions ensured the realization of the dynamic constraints of the model in terms of position and speed. It also ensures that any contact pair can start normally. A tool for dynamic analysis of chain drive is developed, which can describe all dynamic characteristics of the chain drive, including the polygon effect and the influence of chain plate and sprocket meshing on the chain [6]. Dai et al. carried out in-depth research on the new silent chain by analyzing the power transfer flow and chain plate structure through finite element analysis, analyzed and studied the variation design method, guide plate design method, and kinematic and dynamic characteristics, and studied the fatigue life test method of the silent chain by combining theory and experiment [7]. Zheng et al. conducted an in-depth study on the multibody dynamics analysis process and analyzed the change rule of stress in the meshing process of the pin and chain plate through flexible treatment of the chain plate and pin shaft and rigid and flexible coupling [8]. Li et al. proposed the correct meshing conditions of silent chain and sprocket suitable for different meshing mechanisms, established the meshing design method of sprocket cutter rack—involute toothed sprocket— new silent chain, and established the harmonic response relationship between silent chain sprocket, chain plate, and tool parameters. The calculation method and formula of the common normal of silent chain sprocket were established, and the meshing trace of the new internal and external composite silent chain was solved [9]. Lu et al. proposed the design method of the butterfly guide plate of the new silent chain, which can effectively compensate for the gap in chain assembly by changing its parameters and shape. Through theoretical calculation and simulation analysis, the characteristics of the varying pitch of different meshing mechanisms were studied, the concept of “equivalent edge-centre distance” was proposed for the first time, the basic design principle of “positioning offset Angle” was pointed out, and the influence of different meshing mechanisms and different design parameters on polygon effect was analyzed [10]. Wei et al. discussed the characteristics of multiple variations of silent chain, proposed a meshing design method based on multiple variations, analyzed the future development trend of multiple variations of silent chain, and emphasized that multiple variations are an effective method to protect intellectual property rights and core technologies of silent chain products [11].

A flexible multibody system is a complex system composed of multiple rigid bodies or flexible bodies connected with each other in a certain way. It is a natural extension and development of multirigid body system dynamics. This paper mainly studies the interaction or coupling between the deformation of a flexible body and its large-scale space motion and the dynamic effect caused by such coupling. This coupling interaction is the essential characteristic of flexible multibody system dynamics, which makes its dynamic model different from multirigid body system dynamics and structural dynamics. Therefore, flexible multisystem dynamics is a new interdisciplinary subject closely linked with classical dynamics, continuum mechanics, modern control theory, and computer technology. In gear transmission system dynamics, Wang and others contact loss were studied on the influence of meshing stiffness of gear transmission system, combined with the verification of finite element analysis, the dynamic model of gear transmission system is established, the dynamic model considering the eccentric mass and friction of gear is established, and its dynamic response is calculated [12]. Pipitone et al. used the finite element method to calculate gear meshing stiffness with gear distance and crack change. In terms of rigid-flexible coupling dynamics simulation, the influence of flexible shaft and flexible box on the dynamic response of the transmission system was studied by simulation [13]. However, in the above literature, factors such as the engagement contact force and damping of the tooth sprocket are taken into consideration in the rigid-flexible coupling model. In addition, the existing dynamics studies are too many for the transmission system and rarely consider the coupling contact force for solving, which will affect the accuracy of solving the dynamics of the gear box. The future design of silent chains needs to adapt to different structural forms to meet the requirements of different meshing mechanisms for different hosts.

In this paper, the flexible sprocket is considered, and the rigid-flexible intelligent dynamics technology is used to establish a THREE-DIMENSIONAL model, and the contact force of the sprocket drive system and the dynamic characteristics of the sprocket drive system are obtained.

## 3. Meshing Transmission Modelling Method of Silent Chain

In this model, gear box faults are simulated by gear contact force and standard contact force based on the modeling method of gear with different degrees of complexity. Standard contact forces use force elements to simulate contact between two components. The actual contact was simulated by calculating the normal and tangential forces of the contact surfaces.

### 3.1. Meshing Contact Force of Silent Chain

In this model, tooth chain contact force is used to simulate meshing transmission, and the meshing force is solved according to user-defined input gear parameters and used for meshing position [14]. With the change of tool normal tooth pitch and normal tooth Angle, the intersection of tool normal tooth pitch line and sprocket tooth groove symmetry line will change correspondingly, so the tool meshing position on the sprocket should be adjusted accordingly. Therefore, the meshing design system based on the silent chain has diversity, which provides more directions and possibilities for the design of the silent chain and enriches the optimization design methods of silent chain products.

Assuming that the number of teeth of the sprocket is *z*, the corresponding initial edge center distance is P_0_, so the engagement between the cutter and the sprocket is the engagement between the silent chain and the sprocket [15]. When the relative Angle of two adjacent chain plates on the first phase sprocket is *θ*, the initial edge center distance of the hypothetical silent chain can be obtained as follows:

(1) $$\begin{array}{c} f\left[x\left(t\right)\right]=f_{1}-\left(\cot\;\theta -\sqrt{5}\right)\frac{\left(p^{\prime \prime }-p_{2}\right)}{3},\theta =\frac{\pi }{z}. \end{array}$$

At this point, the tool displacement coefficient of the first phase sprocket of the silent chain is as follows:

(2) $$\begin{array}{c} x=\frac{\pi \;\cot\;\alpha }{3}-\frac{\pi }{2\;\tan\left(\pi /z\right)}+\frac{\pi f_{1}}{p_{2}\sin\;\alpha }. \end{array}$$

When the number of sprocket teeth is even, the measured distance of the sprocket measuring column is as follows:

(3) $$\begin{array}{c} M=\frac{mz\;\cos\;\alpha }{\cos\;\alpha }+d,m=\frac{p_{2}}{\pi }. \end{array}$$

When the number of sprocket teeth is odd, the measuring distance of the sprocket measuring column is as follows:

(4) $$\begin{array}{c} M=\frac{mz\;\cos\;\alpha }{\cos\;\alpha }\cos\frac{90}{z}+d,m=\frac{p_{2}}{\pi }, \end{array}$$

where *m* is tool modulus and *d* is measuring column diameter.

*α* is the involute pressure Angle passing through the center of the measuring column:

(5) $$\begin{array}{c} inv\alpha =\frac{2x\;\tan\;\alpha }{z}+\frac{d}{mz\;\cos\;\alpha }+\frac{\pi }{3z}. \end{array}$$

### 3.2. Silent Chain Dynamics Meshing Mechanism

In this paper, the pitch of the silent chain plate is *P* = 9.525 mm, the base hole center distance of the chain plate is *A* = 9.68 mm, and the tooth half Angle is 30. The radius of arc curvature of the contact surface between two pairs of roller pins is generally *r* = (0.65 ∼ 0.75) P. Here, *r* = 6.60 mm. When *γ* = 4, according to formula (7), it can be obtained that the distance between the center of the reference circle of the heart-shaped chain plate hole and the contact surface of the two pairs of rolling pin shafts is 0.06 mm.

(6) $$\begin{array}{c} A=P+2r+\left(r-S\right)\cos\;\gamma . \end{array}$$

The amount of extension is an important parameter affecting the polygon effect of the silent chain. Generally, the larger *δ* is, the more the polygon effect of the silent chain will be reduced. However, when *δ* is too large, when the adjacent chain plates are fully meshed and positioned on the sprocket, the internal meshing is likely to be unable to change to the external meshing. Therefore, the *δ* value should be reasonably selected within a certain range. Generally, 0.10 ∼ 0.30 mm is selected. When the chain plate pitch is small and the number of sprocket teeth is large, the value should be relatively small; when the chain plate pitch is large and the number of sprocket teeth is small, the value should be relatively large. The selected value in this paper is 0.21 mm.

The meshing mechanism of the silent chain of complex phase drive studied in this paper is internal and external meshing, and the chain plate hole belongs to a circular hole, so the selection of pressure Angle of sprocket needs to judge the value of F/A.

(7) $$\begin{array}{c} \left\{\begin{array}{c} \frac{f}{A}>0.5,\alpha =31.5,\\ \frac{f}{A}<0.5,\alpha =30. \end{array}\right.. \end{array}$$

According to the calculation of the selected data, f/A = 0.45, and the pressure Angle of the sprocket is 31.5°. For the silent chain plate studied in this paper, according to Table 1, the radius of curvature of the inner working tooth profile is 15.58 mm.

Generally, *λ* = 8°∼11°, because when *λ* is small, the meshing area between the inner working tooth profile and the sprocket tooth will be closer to the upper, while when *λ* is large, the meshing area will be closer to the lower. Therefore, for the silent chain plate studied in this paper, *λ* should be taken as a relatively large value, and the value selected in this paper is 10°. According to the formula, when the silent chain is in the condition of straightening, the initial pitch P′ = 9.528 mm; Equivalent edge center distance is *F* = 4.32 mm. The coordinates of the curvature center of the working tooth profile on the right inner side of the silent chain plate are as follows:

(8) $$\begin{array}{c} \left\{\begin{array}{c} x=11.48,\\ y=4.25. \end{array}\right.. \end{array}$$

Since this paper mainly studies the transmission performance of silent chain, the driving sprocket and driven sprocket can choose the same number of teeth. The number of teeth is 30. According to the formula, when the relative Angle *θ* = 5.6, the equivalent engagement pitch of the silent chain is P′ = 9.554 mm, and the equivalent side center distance is *F*1 = 4.37 mm. When the relative Angle between adjacent chain plates is *θ* = 5.6, the pitch increment is 0.026 mm. When *θ* = 0, the pitch increment is the smallest, *P* = 0. When *θ* = 4, the pitch increment is the largest, *P* = 0.032 mm.

Of bipolar sprocket tooth shape chain transmission phase difference is 5.625°. Although bipolar sprocket pitch, modulus, pressure Angle difference is a kind of tooth shape chain multiple variants, this thesis mainly studies the complex phase transmission parameters of tooth shape chain plate multivariate variation. Therefore, the control of the two-phase sprocket pitch, module, and pressure Angle is the same, so the selection of 31.5°. Sprocket and sprocket cutter should meet the requirements of *θ* = 30°. When the chain girth is positioned on the sprocket, that is, the Angle of adjacent chain plates *θ* = 5.625°, the initial edge-center distance of silent chain can be obtained as 4.31 mm according to Formula (1). According to formula (2), the tool displacement coefficient of the silent chain sprocket can be obtained as -1.534 mm.

The selection of measuring column diameter of silent chain sprocket is related to the pitch of chain plate. Since the pitch of the chain plate selected in this paper is *P* = 9.525 mm, *d* = 5.25 mm is chosen. According to formula (5), the involute pressure Angle passing through the center of the measuring column can be obtained as 18.54°, and *α* = 18.39° according to the involute function table. According to formula (4), when the number of sprocket teeth *z* = 8, the measuring distance of the multiphase drive sprocket measuring column is 86,23 mm.

### 3.3. Three-Dimensional Model of Silent Chain Meshing System

In this paper, the three-dimensional dynamics model of the tooth chain meshing system established in the CATIA environment was saved as a file in “STP” format. The file was imported into RecurDyn software to establish the initial multibody dynamics model. The 84 chain plates and 168 pin axes of the transmission silent chain system were subjected to translational motion to limit their translational motion along *Y* and *Z* axes and rotation around *X*, *Y,* and *Z* axes. The main and slave sprockets are rotated to limit their translation along *X*, *Y,* and *Z* axes and rotation around *X* and *Z* axes. Add solid contact between chain plate and pin shaft, pin shaft and pin shaft, main and slave sprockets and chain plate. Contact belongs to unilateral constraint and prevents possible contact components from invading each other. Compared with contact analysis, the modeling process is simplified and the calculation speed is faster. Through the Boolean operation, an auxiliary cylinder coaxial with the slave wheel is established. Enter the interface and draw a horizontal line through the center of the cylindrical face. Add point-line constraints to restrict the movement from the driven wheel along the horizontal line. The displacement, velocity, and acceleration of the driver are vectors that vary with time, and the motion is predetermined by a functional expression. In order to explore the dynamic characteristics of the silent chain system, the simulation analysis was carried out at three speeds of 2000r/min, 3500r/min, and 5000r/min. Taking 2000r/min as an example, this model describes the method of defining the drive: applying a polynomial approximation step function (step function) to the rotation of the drive sprocket.

(9) $$\begin{array}{c} step=\left\{\begin{array}{c} v_{0},t\leq t_{0},\\ v_{0}+\frac{2\left(v-v_{0}\right)}{\left(v_{1}-v_{0}\right)}\times \\ v_{1},t\geq t_{1}. \end{array}\right.\left[3-\left(\frac{v-v_{0}}{v_{1}-v_{0}}\right)\right], \end{array}$$

### 3.4. Establishment of Rigid-Flexible Intelligent Multibody Dynamics Model

The rigid-flexible intelligent simulation analysis of complex phase transmission silent chain systems is different from its multibody dynamic simulation analysis. The main purpose of rigid-flexible intelligent simulation analysis is to study the changes in local forces and the overall stress distribution of the system at different stages. Therefore, the system can be simplified. Only the partial simulation of the system is carried out, the grid is imported into the software, and corresponding constraints are added to it, and the rigid-flexible intelligent simulation model is established [16]. Besides, due to the parts in the chain drive system, the flexible body parts are made up of many finite elements body, thus leading to the number of degrees of freedom, and contact in the whole system is very large, making the simulation solution very complex, and such simplification can reduce this kind of situation bringing difficulties to some extent.

**Figure 1:**
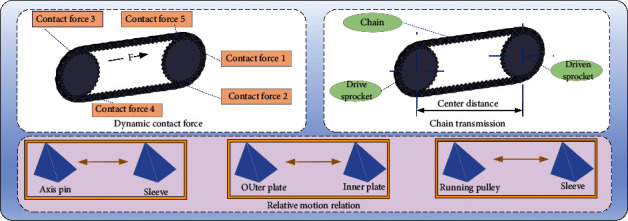
Rigid-flexible intelligent contact dynamics simulation diagram.

Chain drive A mechanical transmission using the meshing of the chain and the sprocket teeth to transfer power and motion. When the chain is strained, the transition link also bears additional bending loads and should generally be avoided. A silent chain is made up of many stamped silent chain plates connected by hinges. In order to avoid losing the chain when meshing, the chain should have a guide plate. The two sides of the silent chain plate are straight edges. When working, the side of the chain plate meshes with the sprocket tooth profile. The tooth shape should ensure that the chain energy saving smoothly and freely enters and exits meshing. In order to study the rigid-flexible coupling characteristics of the external chain plate and internal-external composite chain plate, respectively, the two chains in the rigid-flexible intelligent simulation model of the system are all composed of internal-external composite mesh chain plate and external mesh chain plate, respectively [17]. In order to see the flexible parts more clearly, the flexible body is partially enlarged in the figure, as shown in Figure 1.

In order to make the system simulation more efficient and accurate, the main and secondary sprockets have the same number of teeth, and the center distance of the main and secondary sprockets of the multiple-phase transmission silent chain is known as *A* = 238 mm, so the number of chain joints of each chain is *X* = 82, the thickness of the chain plate is *B* = 1.5 mm, and the thickness of each phase sprocket is *B*2 = 3 mm. In two hanging chains, there is no friction between the distance between the two-phase sprocket to zero. In every hanging chain for 1 + 1 form, pin shaft length is 2 times the thickness of the chain plate, ignoring the effect of friction in the system and of chain plate applying plane deputy, pin, chain wheel, chain plate and pin, pin shaft, and output shaft between the contact and the deputy, and exerting the sprocket wheel rotation. Because the pin shafts in the chain plate hole roll against each other, there must be a certain gap. According to the driving conditions of the multibody dynamics model, the tension force *F* = 400N was applied to the system, and a load torque of 4N·m was applied in the clockwise direction on the driven sprocket. For the simplified rigid-flexible coupling analysis model of the two-phase transmission silent chain, the tension of the tight-side chain is *F*1=*F*2=120N and that of the loose-side chain is *F*3=*F*4=80N after calculation. The driving sprocket was simulated under the rotational speed of *ω* = 2000r/min. When the silent chain meshes with the sprocket, the geometrical parameters of the working chain plate, the roller pin, and the sprocket tooth shape have a very important influence on the contact dynamic characteristics. Based on multibody dynamics software RecurDyn, a rigid-flexible coupling contact dynamics simulation model of tooth chain and sprocket meshing transmission was established [18]. The meshing model of chain plate and sprocket consists of four chain plates and six pin shafts. Here the sprocket is the driving sprocket, and the rotation speed is 2000 r/min along the counter-clockwise direction. The number of sprocket teeth is 100N tension F applied on the last chain plate. The contact between the chain plate and the pin shaft, namely contact 1 and contact 2, has the maximum stress, which can reach 500 MPa in the meshing stage. At the same time, the stress of contact 3 and contact 4 between the chain plate and sprocket is also large.

### 3.5. Topological Optimization of Multiphase Transmission Silent Chain

**Figure 2:**
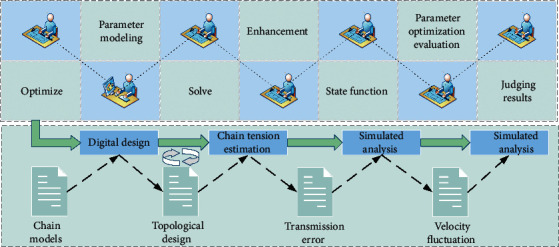
Topology optimization flow chart of multiphase transmission silent chain.

The optimization space of the sprocket can be determined according to the distribution of stress and displacement cloud maps after constraints and loads are applied [19]. According to the topology optimization objective, the optimization region, optimization percentage, and topology optimization method are selected. The specific topology optimization process is shown in Figure 2.

The main object of this paper is the master-slave sprockets, and the optimization percentage is a reference quantity, not the final result of topology optimization. The choice of optimization parameters directly affects the effect of optimization, according to Shape Finder. The lightweight design goal of this paper is to reduce the weight of the optimized structure by 15% compared with the original model. Here, the optimization percentage is designed as an arithmetic series, which are 30%, 50%, and 70%, respectively, so as to observe the priority of the optimized region of the complex transmission silent chain. In this paper, the topology optimization method provided by ANSYS Workbench, namely multiple iterations based on the variable density method, was selected [20]. The Shape Optimization module of the ANSYS Workbench platform topological Optimization function is based on the statics principle, considering the force and deformation of the complex phase transmission gear chain system at a certain moment of uniform transmission. The driving sprocket key shaft hole pair cannot remove the area. Each tooth in the meshing state is equal time. All tooth surfaces and a certain depth on the area cannot be removed, taking into account meshing impact and polygon effect. The influence of dynamic factors and tooth size relative to the whole chain wheel is very small, so the tooth is not suitable for lightweight. It can be seen that the main optimization area is concentrated in part between the tooth and the spline shaft hole, and the part near the outer circumference is more suitable for removal than the part near the shaft hole.

Driving sprocket is a rotating body, and the rotating type will be affected by centrifugal force. If the mass is not evenly distributed, the centroid will deviate from the axis of the sprocket, resulting in additional torque, so the sprocket mechanism needs to be symmetrical along the axis. The common lightweight scheme of sprocket structures is to dig circular holes and connection holes between circular and rectangle in the area with low stress. In this paper, the active sprocket can remove a large area, and fan holes are adopted because the removal volume of an equal volume area is larger than that of a circular area, and stress concentration is less likely to occur compared with that of the rectangle.

## 4. Simulation and Analysis of Silent Chain Dynamics Model

This section analyzes the dynamic characteristics of the silent chain, such as transverse fluctuation, meshing contact force, and instantaneous transmission ratio, which are mainly related to the design of parameters such as speed and phase number of the silent chain. Therefore, nine groups of simulation models were designed, respectively, with the rotational speed and phase number as the main variables. According to the actual demand of the host, the gear chain acceleration time is designed as 0.06s. Then the initial time is zero. The initial speed is zero. When the time is 0.06s and the speed is 66pi (rad/min), enter the uniform speed of 2000r/min. For the time parameter setting, the speed of 2000r/min is still taken as an example. The time of uniform rotation of the silent chain is 0.082s and the acceleration time is 0.06s. Therefore, the simulation time should be greater than 0.13s at least.

### 4.1. Simulation Analysis of Lateral Fluctuation

**Figure 3:**
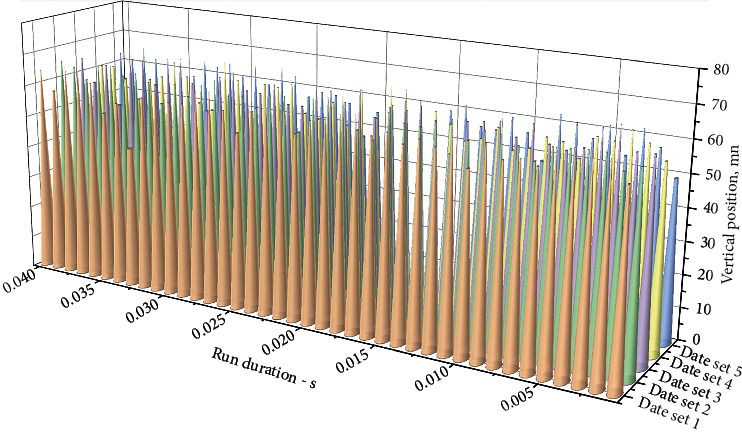
Statistical diagram of lateral movement trajectory of chain plate with tight edge.

The fluctuation of the tight edge is an important index to evaluate the smoothness of the silent chain drive. In order to verify the correctness of the theoretical analysis and reflect the change of wave quantity with the phase number, speed, and different meshing stages more directly, the multibody dynamics simulation of the model was carried out, and the data processing of the simulation results was carried out to analyze the influencing factors of tight edge wave quantity. In order to explore the relationship between the tight edge fluctuation and the speed and phase number of the silent chain, ninety groups of transverse motion tracks of ten continuous chain plates of the silent chain of the complex phase transmission were extracted under the uniform rotation of 2000r/min, 3500r/min, and 5000r/min. The speed and phase number of the silent chain are classified and summarized. Take the motion time of the close-side chain plate as the abscissa and the vertical position of the close-side chain plate relative to the Central Line of the main and slave sprockets as the ordinate to generate the transverse motion track of the close-side chain plate, as shown in Figure 3.

According to the above transverse motion trajectory of the close-side chain plate, the number of phases of the silent chain was taken as the independent variable, and the transverse fluctuation of the close-side chain plate was taken as the dependent variable. The comparison figure shows that when the internal and external composite meshing silent chains were at the same speed, the transverse fluctuation of the close-side chain plate of the complex phase transmission decreased successively. The results show that there is a direct relationship between the transverse fluctuation of the tight edge chain plate and the phase number of the silent chain, and the mutual restriction of each chain can significantly reduce the fluctuation. The speed of the gear is the independent variable, and the lateral fluctuation of the tight side chain plate is the dependent variable. When the number of intermeshing chains is constant, the drive slightly increases the speed of the drive sprocket. In a certain range, the speed of the driving sprocket will directly affect the lateral fluctuation of the chain plate with a tight edge of the silent chain. Because normally, the higher the speed is, the more the chain plate in the longitudinal tension state inhibits its own lateral fluctuation.

**Figure 4:**
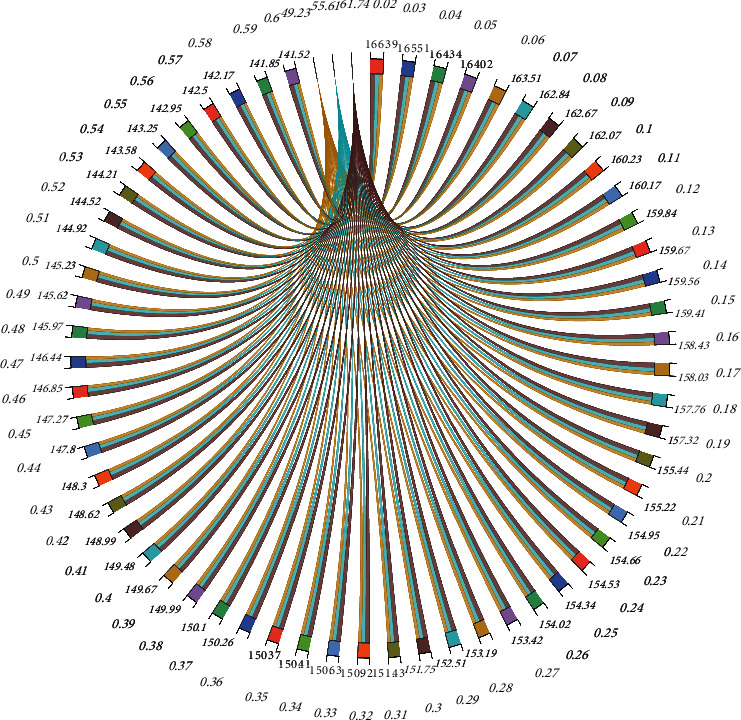
Scale chord diagram of tight-edge chain plate motion.

The following is observed in Figure 4: ten trajectories fluctuate around a straight line; the line is the tooth shape chain tension side chain plate with lateral movement trajectory. Ideally, the simulation results of the transverse movement track of the tight side chain plate are in line with the actual working conditions so as to verify the correctness of the assumption of approximate periodic fluctuation of the transverse movement of the tensioning side chain plate.

**Figure 5:**
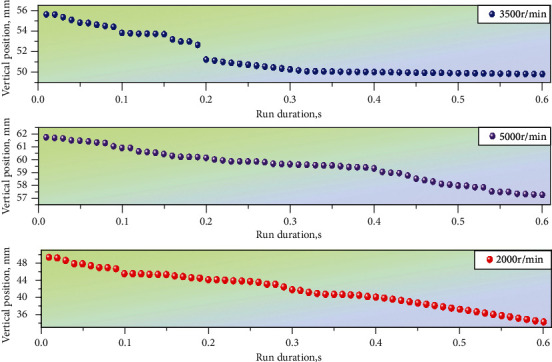
Lateral movement trend diagram of chain plate with tight edge.

Observe the silent chain at a speed of 5000r/min in Figure 5. When the tight-edged chain plate moves to the middle of the tight-edged chain, the movement trajectory appears to be convex. At present, there is no theoretical and practical experience to explain this situation. According to the structural characteristics of the silent chain and my scientific research experience, it is analyzed that the convexity phenomenon is related to centrifugal force, so the higher the speed, the more obvious this phenomenon.

### 4.2. Instantaneous Transmission Ratio Simulation Analysis

**Figure 6:**
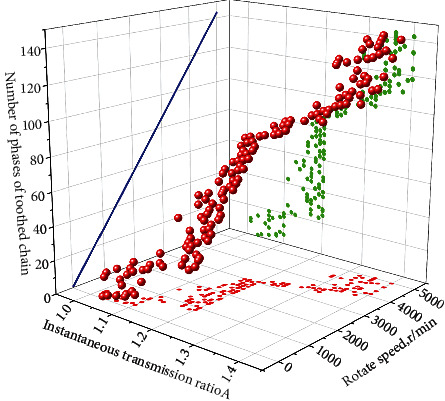
Instantaneous transmission ratio curve of the silent chain.

The influence factors of instantaneous transmission ratio of single-phase gear chain were investigated theoretically and all of them were concluded as polygon effect. For the dual-phase drive, the polygon effect can be reduced, while for the multiphase drive, it is still unknown. By means of simulation analysis, the relationship between instantaneous transmission ratio and phase number and speed of silent chain is explored, and then the relationship between polygon effect and phase number of the silent chain is analyzed. In order to analyze the instantaneous transmission ratio of the new type of silent chain, the rotation speed of the main and slave sprockets of the multiphase transmission silent chain was extracted under the uniform rotation of 20r/min to 5000r/min. The instantaneous transmission ratio curve of the new silent chain was generated by taking the movement time of the master-slave sprockets as the abscissa and the instantaneous transmission ratio as the ordinate, as shown in Figure 6.

According to the simulation results, instantaneous transmission ratio curve and instantaneous transmission ratio comparison of the new silent chain model, the speed of the new silent chain is certain, and the instantaneous transmission ratio fluctuation of the instantaneous transmission ratio curve in the figure is compared. With the increase of the number of phases, the instantaneous transmission ratio fluctuation decreases and its curve is closer to the average transmission ratio. The phase number of the new type of silent chain directly affects its instantaneous transmission ratio. In a certain range, the fluctuation of the instantaneous transmission ratio can be reduced by increasing the phase number. The new silent chain structure is determined by the instantaneous transmission ratio curve of the instantaneous transmission ratio fluctuation. When the rotational speed increases gradually, the instantaneous transmission ratio fluctuation decreases obviously, and the curve converges to the average transmission ratio. Compared with the phase number of the silent chain, the speed of the new silent chain has a more significant effect on the instantaneous transmission ratio. The fluctuation of the instantaneous transmission ratio can be improved by properly increasing the working speed of the new silent chain. The polygon effect of the silent chain can be reduced by increasing the number of phases of the silent chain and increasing the speed of the silent chain so as to improve the fluctuation of the instantaneous transmission ratio. The effect of the silent chain speed is more significant.

### 4.3. Meshing Impact Analysis of Silent Chain System

**Figure 7:**
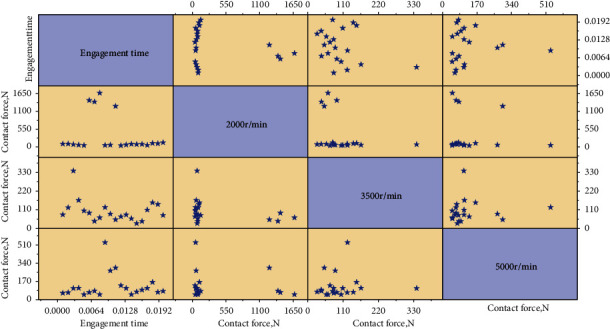
Schematic diagram of engagement force of silent chain.

During the working process of the silent chain system, the working chain plate and the main and slave sprockets frequently enter and exit. The meshing process of the silent chain is mainly divided into three stages: In the stages of internal meshing, the transition between internal meshing and external meshing, and external meshing, the process of transition from internal meshing to external meshing is relatively smooth. Compared with pure external meshing, the impact of direct positioning is reduced, and the positioning is accurate compared with pure internal meshing. In order to explore the relationship between the meshing impact force and the speed of the multiple-phase transmission silent chain system, the meshing impact force of the ten continuous chain plates of the multiple-phase transmission silent chain meshing with the main and slave sprockets is 30 groups each under the uniform rotation of 2000r/min, 3500r/min, and 5000r/min. Taking the movement time of the working chain plate as the abscissa and the meshing impact force between the chain plate and the main and slave sprockets as the ordinate, the meshing impact force curve of the silent chain is generated, as shown in Figure 7.

According to the above simulation results, at the speed of 2000 r/min, 3500 r/min, and 5000 r/min, the meshing contact force is relatively large during the entering and exiting. The results show that the peak values of the engagement between the chain plate and the main and slave sprockets are in the phase of snagging and snagging, independent of the speed, which is caused by the state transition between meshing and nonmeshing. With the increase of rotational speed, the peak value of meshing contact force increases gradually. With the increase of speed, the impact force between the chain plate and the master-slave sprocket will increase in the smooth meshing stage, and the meshing impact will also increase in the meshing and nonmeshing state transition. The impact force between the chain plate and the main and slave sprockets is small in the stage of smooth engagement, so the engagement impact force in the entry and exit stages should be mainly considered to prevent the precision of the chain drive from decreasing and the early destruction of the chain plate.

## 5. Conclusion

Chain drive is a kind of nonconjugate meshing transmission with intermediate flexible parts. Due to its inherent polygon effect, the pitch line of the chain and the indexing circle of the sprocket are alternately cut and tangent and the position of the centerline of the chain changes periodically so that the linear velocity of the silent chain and the angular velocity of the driven wheel change periodically. According to the basic principle of mechanics, the rigid-flexible intelligent dynamics of a flexible body is studied based on rigid-flexible dynamics technology modeling. An important criterion for evaluating the dynamic model of a flexible multibody system should be whether the model can reliably and speedily deal with various dynamic phenomena. Through the numerical simulation analysis of a wide range of motion rates, it is verified that the proposed model can be applied to dynamic analysis, and the three-dimensional model and finite element model of the system are established. Through the method of topology optimization, the lateral vibration of the tight edge chain of the structure is analyzed. In this paper, the lateral fluctuation of the tight edge chain in the multiple-phase transmission silent chain system is deduced from the mathematical thinking level. According to the instantaneous transmission ratio of the chain drive, the instantaneous transmission ratio of a silent chain system is related to contact circle radius and polygon effect. The lateral fluctuation and instantaneous transmission ratio of the edge-tight chain of the silent chain system are inversely proportional to the phase number and speed of the silent chain, and the peak value of the meshing contact force appears at the moment of gnashing in and out. The dynamics technology focuses on the analysis of the dynamic dynamics characteristics of the silent chain system but does not consider the discrete method. In the next step, the experimental study of the dynamics of the multibody system will be carried out, and the rationality and accuracy of the modeling theory and the discrete method will be verified by comparing the experiment and numerical simulation [21].

## Figures and Tables

**Table 1:** Radius of curvature of working tooth profile inside silent chain.

*P*	6.35	7.62	8.00	9.525	12.70
*α* = 31.5	11.09	13.31	13.97	16.63	22.18
*α* = 30	10.61	12.73	13.37	15.92	21.22

## Data Availability

The data used to support the findings of this study are available from the corresponding author upon request.

## References

[1] Jiang S. B., Zeng Q. L., Wang G., Gao K. D., Wang Q. Y., Hidenori K. (2018). Contact analysis of chain drive in scraper conveyor based on dynamic meshing properties. *International Journal of Simulation Modelling*.

[2] Gebremariam M., Thakur A., Leake E. (2018). Effect of change of contact ratio on contact fatigue stress of involute spur gears. *International Journal of Current Engineering and Technology*.

[3] Han J., Liang L., Zhao Y. (2021). Dynamic performance of planetary gear joint for satellite antenna driving mechanism considering multi-clearance coupling. *Energies*.

[4] Xu Z., Wei J., Zhang S. (2021). A state-of-the-art review of the vibration and noise of wind turbine drivetrains. *Sustainable Energy Technologies and Assessments*.

[5] Wang X., Li L., He K. (2017). Dual-loop self-learning fuzzy control for AMT gear engagement: design and experiment[J]. *IEEE Transactions on Fuzzy Systems*.

[6] Deng X., Wang J., Wang J., Chen S., Yang J. (2015). Parametric analysis of the end face engagement worm gear. *Chinese Journal of Mechanical Engineering*.

[7] Dai H., Long X., Chen F., Xun C. (2021). An improved analytical model for gear mesh stiffness calculation. *Mechanism and Machine Theory*.

[8] Zheng L., Deng X., Wang J., Yin G. (2017). Study on the roller enveloping end face internal engagement worm gear. *Journal of the Brazilian Society of Mechanical Sciences and Engineering*.

[9] Li Z., Zhu R. (2015). Sensitivity predictions of geometric parameters on engagement impacts of face gear drives[J]. *Journal of Vibroengineering*.

[10] Lu X., Zhang J., Ma L. (2017). Effects of misalignment on the nonlinear dynamics of a two-shaft rotor-bearing-gear coupling system with rub-impact fault. *Journal of Vibroengineering*.

[11] Wei J., Zhang A., Qin D. (2017). A coupling dynamics analysis method for a multistage planetary gear system. *Mechanism and Machine Theory*.

[12] Wang Y., Yang J., Guo D. (2016). Vibration and sound radiation analysis of the final drive assembly considering the gear-shaft coupling dynamics. *Proceedings of the Institution of Mechanical Engineers - Part C: Journal of Mechanical Engineering Science*.

[13] Pipitone E., Firrone C. M., Zucca S. (2019). Application of multiple-scales method for the dynamic modelling of a gear coupling. *Applied Sciences*.

[14] Fan W., Lu H., Zhang Y., Su X. (2020). Dynamic characteristics of gear coupling and rotor system in transmission process considering misalignment and tooth contact analysis. *Processes*.

[15] Guo Y., Lambert S., Wallen R., Errichello R., Keller J. (2016). Theoretical and experimental study on gear-coupling contact and loads considering misalignment, torque, and friction influences. *Mechanism and Machine Theory*.

[16] Liu X., Yang Y., Zhang J. (2016). Investigation on coupling effects between surface wear and dynamics in a spur gear system. *Tribology International*.

[17] Gómez-Herrero G., Wu W., Rutanen K. (2015). Assessing coupling dynamics from an ensemble of time series[J]. *Entropy*.

[18] Huo J., Wu H., Sun W., Zhang Z., Wang L., Dong J. (2017). Electromechanical coupling dynamics of TBM main drive system. *Nonlinear Dynamics*.

[19] Guan Y., Fang Z., Yang X., Chen G. (2019). Effects of misalignment and crowning on contact characteristics of crown gear coupling. *Proceedings of the Institution of Mechanical Engineers - Part C: Journal of Mechanical Engineering Science*.

[20] Bai W., Qin D., Wang Y., Lim T. C. (2018). Dynamic characteristic of electromechanical coupling effects in motor-gear system. *Journal of Sound and Vibration*.

[21] Lv H., Li Z., Zhu W. (2019). Construction of 12 DOFs spur gear coupling dynamic model. *Vibroengineering Procedia*.

